# The Association between Female Genital Cutting and Spousal HCV Infection in Egypt

**DOI:** 10.1155/2014/164357

**Published:** 2014-03-20

**Authors:** Chris R. Kenyon, Robert Colebunders

**Affiliations:** ^1^Sexually Transmitted Infections, HIV/STI Unit, Institute of Tropical Medicine, Nationalestraat 155, 2000 Antwerpen, Belgium; ^2^Infectious Diseases, University of Antwerp (UA), HIV/STD Unit, Institute of Tropical Medicine, Nationalestraat 155, 2000 Antwerpen, Belgium

## Abstract

*Objective*. To identify the risk factors for HCV infection within married couples in Egypt. *Methods*. In 2008 Egypt conducted its first nationally representative survey of HCV prevalence. 11126 of the 12780 individuals aged 15–59 year who were sampled agreed to participate and provided information via a questionnaire about demographic and behavioural characteristics and blood for HCV antibody and RNA analysis. We assessed the risk factors for HCV infection in a subsample of 5182 married individuals via multivariate logistic regression. *Results*. Overall HCV antibody prevalence in the married couples was 18.2% (95% CI, 16.8–19.6). HCV antibody prevalence was higher in the husbands (23.7%) than the wives (12.1%; *P* < 0.001). Having a spouse who was infected with HCV was an independent risk factor for HCV infection with odds ratios of 2.1 (95% CI, 1.6–2.9) and 2.2 (95% CI, 1.6–3.1) for women and men, respectively. Husbands whose wives had experienced female genital cutting (FGC) had a higher prevalence of HCV and this relationship was driven by a strong association in urban areas. Amongst the women there was no association between FGC and HCV overall but in urban areas only women who had experienced FGC were HCV infected. *Conclusions*. This study provides additional evidence of the importance of intrafamilial transmission of HCV in Egypt.

## 1. Introduction

With 14.7% of 15–59-year-olds testing anti-HCV positive, Egypt has the highest HCV prevalence in the world [1]. Although parenteral antischistosomiasis therapy (PAT) was important in the genesis of Egypt's HCV epidemic this was stopped over 25 years ago and HCV incidence remains high estimated between 150 000 and 500 000 new infections per year [2–4]. Infection from inadequate sterility of dental and medical devices has been shown to play a role in this regard [1, 2, 5–12]. Intrafamilial transmission is an alternative explanation [6]. Support for this theory comes from studies such as a longitudinal study of incidence in two villages in Egypt, which found that the strongest predictor of incident of HCV was having an anti-HCV positive family member [13]. Among those that did and did not have a family member infected with HCV, HCV incidence was 5.8 and 1.0/1000 person years, respectively. Parenteral exposure increased the risk of HCV but was not statistically significant.

This elevated risk of incident of HCV of family members could be due to sharing of implements such as razors or toothbrushes or due to sexual transmission between family members [14, 15]. Alternatively, the elevated risk may be due to shared risk factors (such as the family members all attending a particular health practitioner) rather than being caused by direct transmission between family members [13].

To disentangle these relationships it would be useful to know how HCV is patterned within families. If a husband, is infected is this associated with an increased risk of his wife being infected and vice versa? Is the risk higher for a spouse than nonspousal family members? Are these relationships affected by whether the affected individuals are HCV RNA as opposed to antibody positive?

In 2008 Egypt conducted its first nationally representative survey of HCV prevalence—the 2008 Egyptian Demographic and Health Survey (EDHS). A recently published analysis of this survey found that HCV prevalence increased steadily with age but more so in men than women, reaching, in the 50–59-year-age group, 46.3% in men and 30.8% in women [1]. HCV was also more prevalent in rural than urban areas and on multivariate analysis it was found to be associated with male sex, age, poverty, past history of PAT, and blood transfusion. In urban regions, those with a lack of education and females with genital cutting were more likely to be HCV infected.

This analysis did not however examine the extent to which HCV infection covaried within couples and families. The EDHS is the first HCV survey in the world that is both nationally representative and done in a way which enables researchers to link the HCV status of husbands and wives. In this paper we assess the correlates of HCV infection in 2591 married couples from the EDHS.

## 2. Materials and Methods

**Figure 1:**
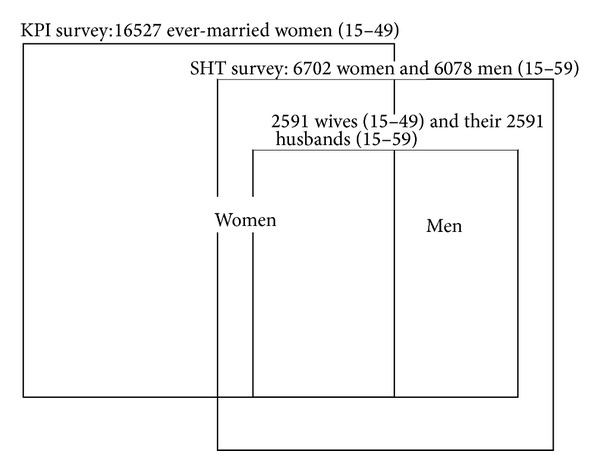
The structure of the Egyptian DHS 2008 and the derivation of the married couples subsample. 16527 ever-married women aged 15–49 were sampled in the key population indicators (KPI) survey. In a subsample of households surveyed in the KPI, 6702 women and 6078 men aged 15–49 were sampled in the special health topics (SHT) component. 2591 wives (aged 15–49) and their 2591 husbands (aged 15–59) could be linked to generate the married couples subsample.

The EDHS entailed a three-stage probability sample that provided a nationally representative sample of 16527 ever-married women aged 15–49 who were interviewed about a range of key population indicators (KPI). In addition, in a subsample of 4953 households, 6702 women and 6078 men aged 15–59 were sampled for a special health topics (SHT) component (see Figure 1). The overall response rate for this latter section was 96.2% and 87.6% for the men and women, respectively. 11126 (87.1%) of these agreed to provide blood for HCV testing. This SHT component was selected so as to provide a sample which was representative for Egypt and the six major areas that the EDHS was stratified by: Urban and Frontier governorates and Upper and Lower Egypt (each of the latter two was divided into rural and urban areas). In order to link husbands and wives, we made use of the fact that 3877 women completed both questionnaires. These were the women who had ever been married, were 14–49 years old, and completed the KPI questionnaire. If the respondent was currently married, then the KPI questionnaire specified the husband's line number within their house. This provides a unique identifier for each husband. Via this mechanism, we established that, in the case of 2591 individuals, the husband of a respondent also completed the SHT component of the EDHS. In this paper we study the relationships between HCV and various risk factors in these 2591 husband-wife pairs. Apart from the 5182 individuals in this married subgroup, further 2338 persons aged 15–59 years, living in the same houses as the married couples, were included in the SHT survey. Although the outcome variable used in this study is the presence of anti-HCV antibodies in the 5182 members of the married subgroup, the relationship between the HCV serostatus of the married couples and that of the other household members is also of relevance. We therefore included the HCV antibody and RNA status of these 2338 individuals as exposure variables in our analyses. We also calculated the HCV prevalence for each of Egypt's 26 governorates. These were used as a measure of local or community HCV prevalence.

Unless otherwise stated the terms “HCV prevalence” and “infection” refer to HCV antibody prevalence. The HCV antibody prevalence rates were calculated for a range of potential risk factors available in the special health topics questionnaire. Because of the strong association between age and HCV prevalence all the odds ratios and *P* values given are age-adjusted. Logistic regression was used to explore the strength of the association of each variable with HCV infection in the 5192 individuals in the married couples cohort.

Tests for interaction between variables were conducted. These tests revealed that the effect of several of the variables varied according to urban/rural location and men/women. As a result, separate models were constructed for men and women as well as urban and rural areas. All the urban women who had not undergone female genital cutting were HCV negative. To avoid the collinearity that this created in the analyses, for the analysis limited to urban women, we randomly selected one urban woman who had not undergone female genital cutting and changed her HCV status as positive. The final models were constructed by including all variables with *P* values <0.2 on univariate logistic regression. The education variable was not included due to significant collinearity with the income variable. The HCV status of the spouse and that of the other household members (both exposure variables) were represented by HCV RNA instead of HCV antibody positivity in the multivariate models due to exerting a stronger effect on the outcome variable (and considerable collinearity between the RNA and antibody HCV tests). All analyses were weighted to account for the sampling and survey design. Statistical analysis was conducted using STATA version 12.0 (StataCorp, College Station, TX).

The HCV prevalence rates for the husbands and wives were also stratified by the wives' excision status to explore how HCV prevalence in both husbands and wives varies according to the excision status of the woman. The terms excision and female genital cutting (FGC) are used synonymously in the paper. The FGC variable was defined as follows: both the women who had experienced FGC and the men whose wives had undergone FGC were coded as 1 and the women and men whose wives had not undergone FGC were coded as 0. To assess the impact of whether HCV prevalence in women was associated with who conducted the FGC, a second FGC variable, termed FGC-operator, was constructed as follows: women with no history of FGC coded 0, FGC performed by doctor and nondoctor coded as 1 and 2, respectively. The multivariate models for women were run separately with the FGC and FGC-operator variables.

A third generation enzyme-linked immunosorbent assay was used to detect HCV antibodies (Adaltis EIAgen HCV Ab, Casalecchio di Reno, Italy). Positive tests were confirmed by a chemiluminescent microplate immunoassay (CIA). Seropositive specimens were tested for HCV RNA using the RealTime_m2000 system (Abbott Laboratories, Abbott Park, IL, USA). Full details of the survey and sampling strategy have been previously published [1, 16].

## 3. Results

Overall HCV antibody prevalence in the married couples was 18.2% (95% CI, 16.8–19.6). HCV antibody prevalence was higher in the husbands (23.7%) than the wives (12.1%; *P* < 0.001; see Table 1). Restricting this analysis to the 15–49-year-olds reduced the difference in HCV between the husbands and wives (18.8% and 11.6% resp. *P* < 0.001). HCV prevalence was also higher in rural (20.4%) than urban (12.0%) regions (*P* < 0.001). HCV prevalence increased steadily with age reaching 30.2% (95% CI, 26.8–33.8) in men and 23.9% (95% CI, 20.4–27.7) in women in the 41–49-year-old category. Amongst women, there was a stepwise increase in HCV prevalence with increasing number of children: 6.9% if 0–2 children, 14.1% if 3–5 children, and 24.5% if more than 5 children. There was a lower HCV prevalence in those who had completed secondary level education (14.3%) compared to those with no education (23.5%; *P* = 0.001) and those in the top two income quintiles (12.1 and 12.9%) compared to those in the poorest quintile (22.8%; *P* < 0.001). HCV prevalence in persons who had received PAT (32.1%) was higher than in those who had not (16.5%; *P* < 0.001). Women with excision had a trend to higher HCV prevalence (12.5%) than those without (3.9%; *P* = 0.096; see Tables 1 and 2). Men whose wives had been excised had a higher HCV prevalence than those whose wives had not (23.7% versus 8.3%; *P* = 0.003). Women who had been excised by a doctor had a lower HCV prevalence than those excised by a nondoctor (5.6% versus 13.7%; *P* = 0.003). Respondents who had received a blood transfusion had nonsignificantly higher HCV prevalence rates than those who had not (26.9% versus 17.8%; *P* = 0.132). HCV prevalence increased with length of marriage, increasing from 8.2% to 17.6 and 29.6% in those married for ten years or less, 11–20 years, and over 20 years, respectively. Having received injections and dental treatment were not associated with HCV seropositivity.

Persons with an HCV seropositive partner had a higher HCV prevalence than those who did not (32.6% versus 15.1%; *P* < 0.001). This effect was also evident if one's partner was RNA positive for HCV (34.7% versus 15.9%; *P* < 0.001). The effect was not as marked if it was another member of the household who was HCV antibody (23.3% versus 17.7%; *P* = 0.004) or RNA positive (23% versus 17.9%; *P* = 0.015).

In the multivariate logistic regression analyses, three variables were associated with HCV infection in all models; see Table 3. These were age, local HCV prevalence, and having a spouse who was infected with HCV. Having a nonspousal household member who was HCV infected was not independently associated with HCV. For both men and women HCV was less prevalent in the richer quintiles but in the case of men this effect was evident in the urban but not the rural areas.

HCV was associated with a blood transfusion in women but this association only applied to the rural areas. PAT was associated with HCV in all the models except in the men in the rural and the women in the urban areas. Being married for longer than 20 years was associated with HCV, but only for men. FGC was associated with HCV infection in the men but not the women overall. This relationship in men was driven by a relatively strong association in urban areas. Amongst the women, there was no association between FGC and HCV overall but in urban areas none of the women who were not excised were HCV infected. In the second set of women's models substitution of the FGC variable with the FGC-operator variable had little effect. FGC had no effect in rural areas and a strong effect in urban areas regardless of whether it was conducted by a doctor or nondoctor (data not shown).

The EDHS reveals that, of the women who had undergone FGC, 99.9% had done so by the age of 18. Of the women aged 15–18 surveyed in the EDHS, the HCV prevalence was significantly higher in those who had been excised (39/723; 5.4%) than those who had not (0/164; 0%, *P* = 0.028).

There was no evidence of interaction between the wife's FGC status and HCV status of the partner variables.

## 4. Discussion

Linking husbands and wives allowed us to test the association of HCV infection between husbands and wives. This represents the first time that this has been done in a nationally representative HCV survey. The sampling strategy used to describe the epidemiology of HCV in the USA, although nationally representative, does not include sexual partners in a linked way that would allow a similar analysis [17]. We found an association between the HCV status of the respondent and their partner. This is true for analyses limited to rural and urban areas and for subanalyses of men and women within these areas. The association remains after controlling for other members of the household being HCV infected. The relationship is slightly stronger when the HCV in the partner is measured with an RNA-based as opposed to an antibody-based test.

If not due to confounding, this association may be due to nonsexual intrafamilial transmission (such as shared utensils, toothbrushes, and razors), sexual transmission, or shared risk exposures (such as attending to the same health care practitioner). If the former was predominant then we should expect an association between HCV infection in nonspousal family members and in respondents. There was no evidence of such an association in any of the multivariate models. In our models we controlled for a large number of plausible, shared risk exposure types (such as blood transfusions, multiple injections, and PAT), but these did not affect the strength of the relationship between respondent and partner HCV status. The stronger association between the respondent's HCV status and that of their wife/husband as opposed to that of other family members may be mediated by the greater length of time they spent together. The fact that there is a relationship between length of marriage and HCV infection (for men) could be interpreted as supporting evidence for this idea. It does not however explain why this relationship only applies to men. An alternative explanation, and one that is also supported by the relationship between HCV infection and length of marriage, is that sexual transmission between partners is responsible for the relationship of HCV infection in married couples.

We cannot however exclude the possibility that the reason why the association between the wife and the husband's HCV status remains strongly positive after controlling for the HCV status of the other household members is due to the partner's HCV status being a better measure of general (nonsexual) infection pressure than the HCV status of the other household members. In the models we do control for the HCV in the surrounding community, but this is defined at the level of the governorate. This may not be a local enough measure of community HCV prevalence.

The relationship between HCV and FGC is complex. There is a strong relationship between HCV infection and FGC in the urban areas but none in the rural areas. There was little sex-based difference. For the men in the urban areas there is an association between HCV infection and having a wife who was excised (OR 3; 95% CI, 1.1–7.9). In the case of women, none of the nonexcised women had HCV infection.

How do we explain the discrepancy between the rural and urban areas? One possibility is that circumcision in urban areas is more likely to transmit HCV. Though this is possible, it should be noted that it is circumcision by nondoctors that is most strongly correlated with HCV infection [18] and in rural areas the proportion of FGC performed by nondoctors is higher (84.2%) than in urban areas (73.0%; *P* < 0.001) [18]. Another possibility is that FGC is so prevalent in the rural areas (97.2%) that there are too few nonexcised women to be able to demonstrate an effect of FGC on HCV prevalence. For example, in two of the other studies, to consider the impact of FGC on HCV in Egypt, no effect was found, but this may have been due to the extremely low numbers of persons not excised. In the first study there was only one person (out of 1989 individuals in the survey over the age of 20) who was not excised [5]. In the second study, only 4 women out of 1051 (0.4%) over the age of 30 were not excised. This study found a nonsignificant increase in the risk of HCV infection in those women who had been excised by an informal health care provider as opposed to those nonexcised combined with those excised by a formal health care provider (OR 1.6; 95% CI, 0.7–3.8). In a separate analysis of the EDHS a strong ecological association was found between the prevalence of FGC and HCV at the governorate level [18].

FGC has been associated with range of infections [19]. A population-based, cross-sectional study from the Gambia, for example, found a strong association between prevalent FGC and herpes simplex virus-2 infection (OR 4.7, 95% CI, 3.7–6.4) and a weaker association between FGC and bacterial vaginosis [20]. A case-control study of primary infertility in Sudan found more extensive forms of FGC to be more prevalent in the cases [21]. There were too few cases and controls without FGC in Sudan study to allow any analysis of those with versus those without FGC. The evidence from Egypt is mixed. A case control study of the determinants of infertility found that cases were more likely to have been excised by a traditional practitioner and more likely to have had more extensive forms of FGC [22]. A later study found no association between FGC and infertility [23].

What could be the possible mechanisms for FGC to result in increased rates of HCV for both men and women? Inadequate sterilization of implements used to perform FGC could be a factor. The higher HCV prevalence in excised versus nonexcised 15–18-year-olds in the EDHS could be interpreted as evidence supporting this nonsterility hypothesis. In addition, HCV transmission at the time of FGC could have been greater in the past when a considerably greater proportion of FGC procedures were performed by nondoctors [18]. Two studies from Egypt have found an association between male circumcision performed by informal health care providers and prevalent HCV infection [5, 24].

The anatomical changes produced by FGC, particularly the more extensive forms of FGC, could also promote subsequent female to male and male to female HCV transmission. It is biologically plausible that FGC could both enhance women's susceptibility to the sexual transmission of HCV and increase the chances that HCV is transmitted to their partner [23]. We cannot however exclude the possibility that the relationship between FGC and HCV is due to an unmeasured confounding variable.

There is considerable controversy in the literature about the extent to which HCV can be transmitted by sexual contact and cohabitation. In general most studies and two systematic reviews have found that HCV can be transmitted sexually but that the risk of infection is low [14, 25]. The most recent systematic review tried to make sense of the conflicting results by dividing the studies into those from high (Japanese) and low (non-Japanese) prevalence regions [14]. They found that pooling the results of studies along these lines provided strong evidence of increased HCV prevalence in offspring of affected persons in endemic areas but no such effect in nonendemic areas. In contrast they found evidence of an increased HCV prevalence amongst the spouses of persons who were HCV seropositive in nonendemic areas but no evidence for this effect in endemic areas. One interpretation of these apparently discordant findings is that HCV prevalence in spouses of HCV seropositive persons was not higher than controls in endemic areas as the prevalence in the controls was so high [14]. In endemic settings, transmission rates may be so high that close to all susceptible persons are infected by the time they are married. This may mask any effect that domestic and sexual transmission may play. An analogous effect was observed with hepatitis B virus. Sexual transmission was shown to occur in low prevalence areas such as USA but not in high prevalence areas such as East Asia [26, 27]. More recent studies have found evidence of spousal transmission of HCV in endemic areas [5, 6, 11, 13, 28–30]. Genotypic studies provide further evidence of the spousal transmission of HCV [8, 15, 30, 31].

In Egypt there is an increasing amount of evidence that intrafamilial transmission is an important source of new infections [5, 13, 29]. Two prospective studies investigating the correlates of incident of HCV in Egypt have found evidence of intrafamilial transmission [13, 32]. One of these was a study that followed up a cohort of 6734 HCV antibody negative persons from 2 rural villages over a median of 1.6 years [13]. In this time there were 33 new HCV infections, 27 of which occurred in families with an anti-HCV positive member. Parenteral factors were not associated with an elevated HCV incidence and in 21 of the cases there was no history of any parenteral exposure. HCV incidence per 1000 person years was higher in spouses of HCV antibody positive as opposed to antibody negative persons (13.1 versus 1.9; *P* = 0.08). Men and women with anti-HCV positive spouses were 7 and 2 times as likely to seroconvert as those with seronegative spouses. HCV incidence in children increased in a stepwise manner if they had one of two parents who is HCV antibody positive. A number of other studies have found marriage to be a risk factor for HCV infection but not all of these are controlled for age, which is likely a significant confounder [5, 6, 29]. One study found that parenteral factors only play a part in explaining prevalent cases in those over the age of 20 in Egypt [6].

One way of tying together the seemingly discordant findings about the extent of intrafamilial HCV transmission from different studies around the world is to apply the insight from hepatitis B virus epidemiology that the predominant mode of transmission may vary considerably between different regions of the world. Hepatitis B transmission in East Asia is predominantly perinatal, in USA it is largely sexual and intravenous drug use [33, 34], and in sub-Saharan Africa an important cause is horizontal transmission between children through poorly defined mechanisms [35–37].

In USA, iatrogenic and intravenous drug usage have been shown to be the dominant modes of HCV transmission [17]. There is mounting evidence that sexual transmission is important in HCV outbreaks of men who have sex with men [38]. The best quality evidence however suggests that sexual transmission has not played a large role in HCV transmission among heterosexuals in the USA [17, 39].

The composite evidence from Egypt reveals a somewhat different epidemiology for HCV. PAT was clearly important in the initial amplification of HCV in Egypt [3]. What perpetuated the spread of HCV thereafter? Perinatal transmission can take place. However, most individuals infected by this route clear the virus spontaneously [6, 40]. Unsterile procedures have clearly played an important role [1, 2, 6–8, 11]. A large proportion of cases are however not explained by these factors [1, 6, 9, 13]. Our study backs up the evidence from elsewhere of the likelihood of horizontal spread between family/household members [5, 9]. Some of this may be sexual but much is likely to be via other, as yet unclearly defined, mechanisms [13]. The findings presented here also build on the evidence from elsewhere [1] that FGC may have played a role in the spread of HCV—both at the time of the procedure and via enhancing the sexual transmission of HCV.

This analysis has a number of serious limitations. The EDHS was a cross-sectional survey and thus the direction of any implied causation cannot be established. Only 5182 individuals (out of 11126 individuals surveyed in the special health topics sample) could be linked together to provide the wife-husband dyad sample used for this analysis. Furthermore the limitations imposed by the linking process meant that the ages of the husbands were from a wider age-band (15–59 years old) than that of the wives (15–49). Because of these limitations, the sample we used cannot be assumed to be representative of whole Egypt.

The uni- and multivariate analyses of the married couple subsample are, however, remarkably similar to those found in analyses of the entire sample of 11126 respondents (presented in Guerra et al. [1]). This suggests that our subsample is not significantly biased.

Given the ongoing high incidence of HCV in Egypt [2], further research is needed to better define the mechanisms for intrafamilial spread so as to guide new prevention strategies. In particular further research is needed to ascertain if FGC is an effect-modifier in the sexual transmission of HCV.

## Figures and Tables

**Table 1:** HCV seroprevalence and age-adjusted odds ratios for selected characteristics (Egyptian DHS 2008).

Risk factors	Number of exposed (%)^a^	Number of HCV antibody positive (%)^b^	Age-adjusted OR (95% CI)	*P* value (age-adjusted)
Place of residence				
Rural	3234 (37.6)	661 (20.4)	2.4 (1.9–3.0)	<0.001
Urban	1948 (62.4)	233 (12.0)	1	
Region				
Urban governorates	610 (11.8)	76 (12.6)	1	
Lower Egypt—urban	554 (10.7)	69 (12.3)	1.0 (0.7–1.5)	0.994
Lower Egypt—rural	1608 (31.0)	364 (22.9)	2.7 (1.9–3.8)	<0.001
Upper Egypt—urban	600 (11.6)	79 (13.5)	1.1 (0.7–1.7)	0.778
Upper Egypt—rural	1524 (29.4)	294 (20.1)	2.1 (1.5–3.0)	<0.001
Frontier governorates	286 (5.5)	12 (4.5)	0.3 (0.2–0.6)	0.001
Gender				
Women	2591 (50)	300 (12.1)	0.7 (0.6–0.9)	<0.001
Men	2591 (50)	594 (23.7)	1	
Men's age (years)				
15–20	9 (0.4)	0 (0)		
21–30	524 (20.2)	50 (9.5)	1	
31–40	877 (33.9)	117 (13.3)	1.5 (1.0–2.1)	0.034
41–49	827 (31.9)	268 (32.4)	4.5 (3.3–6.3)	0.000
50–59	354 (13.7)	159 (44.9)	7.7 (5.4–11.1)	0.000
Women's age (years)				
15–20	154 (5.9)	5 (3.3)	1	
21–30	1018 (39.3)	60 (5.9)	1.8 (0.7–4.7)	0.188
31–40	860 (33.2)	104 (12.1)	4.1 (1.6–10.2)	0.002
41–49	559 (21.6)	131 (23.4)	9.1 (3.6–22.7)	0.000
Educational attainment				
Secondary completed	2424 (46.8)	282 (14.3)	0.7 (0.6–0.9)	0.001
Incomplete secondary or less	1473 (28.4)	282 (19.9)	0.9 (0.7–1.1)	0.219
No education	1285 (24.8)	330 (23.5)	1	
Wealth index quintile				
Richest	1010 (19.5)	118 (12.1)	0.4 (0.3–0.5)	<0.001
Rich	942 (18.2)	112 (12.9)	0.5 (0.4–0.7)	<0.001
Middle	1128 (21.7)	225 (21.2)	0.9 (0.7–1.2)	0.413
Poor	1056 (20.4)	220 (22.0)	0.9 (0.7–1.2)	0.600
Poorest	1046 (20.2)	219 (22.8)	1	
Parenteral antischistosomiasis therapy				
No	4582 (88.4)	707 (16.5)	1	
Yes	600 (11.6)	187 (32.1)	1.7 (1.4–2.2)	<0.001
Women: reports FGC^c^				
No	132 (5.1)	4 (3.9)	1	
Yes	2459 (94.9)	296 (12.5)	2.9 (0.8–10.1)	0.096
Men: his wife reports FGC^d^				
No	132 (5.1)	11 (8.3)	1	
Yes	2459 (94.9)	583 (23.7)	3.1 (1.5–6.6)	0.003
FGC performed by^g^				
Doctor	386 (15.7)	21 (5.6)	1	
Nondoctor	2073 (84.3)	275 (13.7)	1.4 (1.1–1.7)	0.003
Blood transfusion				
No	4931 (95.3)	832 (17.8)	1	
Yes	244 (4.7)	60 (26.9)	1.3 (0.9–1.8)	0.132
Multiple injections^e^				
No	4301 (83.0)	751 (18.4)	1	
Yes	881 (17.0)	143 (17.1)	0.9 (0.7–1.1)	0.312
Dental treatment^e^				
No	1829 (35.3)	270 (15.3)	1	
Yes	3353 (64.7)	624 (19.6)	1.1 (0.8–1.2)	0.609
Total number of children^h^				
0–2	1098 (42.4)	73 (6.9)	1	
3–5	1162 (44.9)	154 (14.1)	1.2 (1.0–1.6)	0.427
≥6	331 (12.8)	73 (24.5)	2.1 (1.0–2.5)	0.035
Partner is seropositive for HCV				
No	4288 (82.8)	608 (15.1)	1	
Yes	894 (17.3)	286 (32.6)	2.1 (1.6–2.7)	<0.001
Partner is HCV RNA positive				
No	4569 (88.2)	686 (15.9)	1	
Yes	613 (11.8)	208 (34.7)	2.3 (1.7–2.9)	<0.001
Wife is HCV RNA positive				
No	2291 (88.4)	451 (20.4)	1	
Yes	300 (11.6)	143 (47.4)	2.6 (2.0–3.4)	<0.001
Husband is HCV RNA positive				
No	1997 (77.0)	157 (8.3)	1	
Yes	594 (22.9)	143 (24.3)	2.5 (1.9–3.3)	<0.001
Another household member is seropositive for HCV				
No	4760 (91.9)	798 (17.7)	1	
Yes	422 (8.1)	96 (23.3)	1.6 (1.2–2.1)	0.004
Another household member is HCV RNA positive				
No	4896 (94.5)	834 (17.9)	1	
Yes	286 (5.5)	60 (23.0)	1.5 (1.1–2.1)	0.015
Length of marriage				
0–10 years	2430 (46.9)	258 (8.2)	1	
11–20 years	1574 (30.4)	339 (17.6)	1.2 (1.0–1.5)	0.051
>20 years	1178 (22.7)	461 (29.8)	1.4 (1.1–1.8)	0.016

FGC: female genital cutting.

^a^Unweighted percentage.

^b^Weighted percentage.

^c^Numbers for this row are for women only.

^d^Numbers for this row are for men only.

^e^Defined as 2 or more injections reported in the preceding 6 months.

^f^Ever received dental treatment of any sort.

^g^Of all women who report undergoing FGC

^h^The total number of children that women report giving birth to.

**Table 2:** Prevalence of HCV antibodies in 2591 husband-wife pairs, stratified by female genital cutting (FGC) status of the woman (Egyptian DHS 2008).

	Wife's HCV antibody status (%)		
	Negative	Positive	Total^b ^(%)
Wife has undergone FGC			
Husband HCV negative	1721	155	1876 (76.3)
Husband HCV positive	442	141	583 (23.7)
Total^a^ (%)	**2163 (88.0)**	**296 (12.0)**	**2459 (100)**
Wife has not undergone FGC			
Husband HCV negative	119	2	121 (91.7)
Husband HCV positive	9	2	11 (8.3)
Total^a^ (%)	**128 (97.0)**	**4 (3.0)**	**132 (100)**

^a^Row percentages.

^b^Column percentages.

**Table 3:** Factors associated with 5182 husbands and wives testing seropositive for hepatitis C in the 2008 Egyptian Demographic and Health Survey: multivariate logistic regression model results (odds ratios, 95% confidence intervals, and *P* values).

	Women			Men			Urban women			Urban men			Rural women			Rural men		
	Odds ratio	95% CI	P value	Odds ratio	95% CI	P value	Odds ratio	95% CI	P value	Odds ratio	95% CI	P value	Odds ratio	95% CI	P value	Odds ratio	95% CI	P value
*N*	2546			2546			953			953			1593			1593		
Wife had FGC	2.0	0.7–5.1	0.166	2.1	1.1–3.9	0.026	4.0	0.5–31	0.188	3.0	1.1–7.9	0.025	0.9	0.3–2.5	0.85	1.4	0.6–3.3	0.458
Spouse is HCV RNA positive	2.1	1.6–2.9	0.000	2.2	1.6–3.1	0.000	2.4	1.3–4.4	0.005	2.5	1.2–5.1	0.015	2.0	1.4–2.8	0.000	2.2	1.5–3.2	0.000
Wealth index quintile																		
Poorest	Ref																	
Poor	1.2	0.9–1.8	0.260	0.9	0.7–1.3	0.698	1.9	0.5–7.6	0.379	0.2	0.1–0.6	0.002	1.2	0.8–1.8	0.333	1.1	0.8–1.5	0.527
Middle	1.0	0.6–1.5	0.922	1.2	0.9–1.6	0.248	0.9	0.2–3.5	0.855	0.4	0.2–0.9	0.035	1.1	0.7–1.7	0.670	1.4	1.0–1.9	0.063
Rich	0.7	0.4–1.1	0.097	0.7	0.5–0.9	0.017	0.8	0.2–3.1	0.796	0.2	0.1–0.5	0.000	0.8	0.4–1.4	0.378	0.9	0.6–1.5	0.727
Richest	0.5	0.3–0.8	0.003	0.7	0.5–1.0	0.087	0.6	0.2–2.5	0.508	0.3	0.1–0.6	0.001	0.5	0.2–1.1	0.082	1.3	0.7–2.2	0.381
Age^b^	1.1	1.0-1.1	0.000	1.1	1.0-1.1	0.000	1.1	1.1-1.2	0.000	1.1	1.0-1.1	0.000	1.1	1.0-1.1	0.000	1.1	1.0-1.1	0.000
Blood transfusion	1.9	1.1–3.5	0.028	1.1	0.7–1.7	0.604	1.2	0.4–3.3	0.789	1.4	0.8–2.7	0.273	2.3	1.1–4.7	0.025	0.9	0.6–1.6	0.820
Parenteral antischistosomiasis therapy	1.7	1.1–2.8	0.016	1.3	1.0–1.7	0.025	1.9	0.6–5.8	0.254	2.3	1.4–3.8	0.002	1.7	1.1–2.9	0.028	1.1	0.9–1.5	0.412
Another household member is HCV RNA positive	0.8	0.5–1.4	0.455	1.2	0.8–1.9	0.361	1.1	0.3–4.1	0.915	1.5	0.5–4.6	0.518	0.7	0.4–1.4	0.319	1.2	0.7–1.9	0.496
Length of marriage																		
0–10 years	Ref																	
11–20 years	1.0	0.7–1.6	0.887	1.3	1.0–1.8	0.044	0.6	0.2–1.5	0.285	1.4	0.8–2.3	0.243	1.2	0.7–2.1	0.416	1.4	1.0-2.0	0.056
>20 years	1.0	0.5–1.9	0.941	1.6	1.1–2.4	0.019	0.6	0.2–1.9	0.355	1.5	0.7–3.0	0.264	1.2	0.6–2.6	0.601	1.7	1.0–2.8	0.036
Local HCV prevalence^c^	1.1	1.1-1.1	0.000	1.1	1.0-1.1	0.000	1.1	1.0–1.2	0.001	1.0	1.0-1.1	0.033	1.1	1.1-1.1	0.000	1.1	1.0-1.1	0.000
Number of children^d^	1.0	0.9–1.1	0.674	NA			1.0	0.8–1.3	0.695		NA		1.0	0.9-1.0	0.347	NA		

NA: not applicable.

^a^Not entered into model due to collinearity; all nonexcised women were HCV negative (see text).

^b^Age is defined continuously in years.

^c^Local HCV prevalence is defined as the HCV prevalence in the surrounding governorate.

^d^The total number of children that women report giving birth to, here defined continuously.
